# Supplementary figures and images for: Calcification of the thoracic aorta on low-dose chest CT predicts severe COVID-19
Source: PLoS One. 2020 Dec 23;15(12):e0244267. doi: 10.1371/journal.pone.0244267 (PMC7757863; doi:10.1371/journal.pone.0244267)

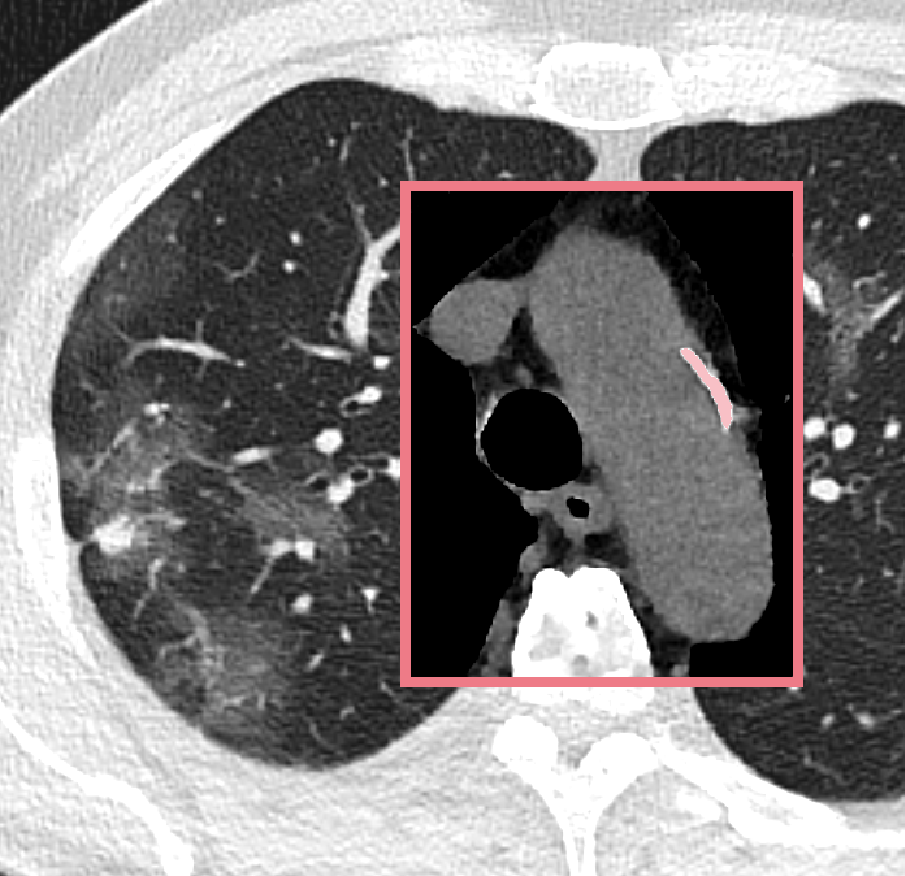

Supplement: S1 Fig — Soft tissue and lung window of an axial computed tomography are combined to illustrate the typical pulmonary findings of COVID-19 besides calcification of the thoracic aorta (light red). (TIF) [file pone.0244267.s002.tif]
