# Supplementary material for: Calcification of the thoracic aorta on low-dose chest CT predicts severe COVID-19
Source: PLoS One. 2020 Dec 23;15(12):e0244267. doi: 10.1371/journal.pone.0244267 (PMC7757863; doi:10.1371/journal.pone.0244267)
Supplement: Attachment — Submitted filename: Rebuttal letter.docx [file pone.0244267.s003.docx]

Comments of Reviewer #1:

-The language employed in the abstract is unclear. The authors note that 32/24 patients presented as mild/severe COVID-19, but state earlier that CT was performed to assess the risk of severe COVID-19 and that subsequent outcomes were rated as mild or severe based on factors such as hospitalization, ICU, intubation, etc. Please clarify whether the severity of COVID-19 being utilized in the model is patient outcome, or severity at presentation.

*Outcome was rated by clinical endpoints which were moderate (no hospitalization, hospitalization) or severe (admission to ICU, tracheal intubation, death), see ll. 26-28. The worst clinical endpoint during the observation period was noted, see ll. 119-121.*

-Did AWC provide any additional predictive benefit of COVID-10 severity beyond predictions based off of the severity of pulmonary changes from LDCT (e.g. ground-glass opacity sub-type, lung opacification mass, number of zones involved, grading, etc)? It does not appear that LDCT pulmonary changes were quantified nor their predictive value assessed as a control comparison, why not?

*Quantification of lung involvement has been successfully correlated with short-term 5-day outcome of COVID-19. However, in early stage the disease evolves most rapidly and the involvement of the lung above all depends on the time point of imaging, see ll. 228-232. Hence for long-term outcome we examined the more stable parameter of AWC which doesn’t fluctuate during early course of the disease.*

-Patient age is likely to be highly correlated to both degree of cardiovascular calcification and, based on literature reports to date, severity of COVID-19. Patient age appears to provide little additional predictive benefit of COVID-19 severity beyond AWC. Is AWC effectively a surrogate for patient age in this population?

*After including a larger patient population we could show that the magnitude of AWC was the only significant regressor of severe COVID-19 progression. Control variables such as patient age and sex were no significant regressors, see ll. 186-188. Comorbid patients generally develop more severe COVID-19. Our findings appreciate that magnitude of AWC illustrates a more comprehensive status of patients’ comorbidities than simple biographical age, see ll. 243-251.*

-Please include specific Wilcoxon test p values in Table 1 for each factor. Why was a paired non-parametric test used on continuous data such as patient age, AWC, and Agatston score?

*Table 1 was completed by the p-values for patient age, sex and AWC, see l. 174. Statistical tests were revised.*

-Was predictive power significantly improved in men vs. women? Why are AWC predictive AUC values higher in women than in men?

*Gender specific analysis was omitted after revision of the statistical methods, since patient sex was no significant regressor for severe COVID-19.*

-Patient populations are relatively small for this sort of predictive modeling with such scaled severity measures; were power analyses performed to ensure the appropriate-sized study group?

*In contrast to the ROC analysis of the preliminary manuscript, AWC was a highly significant regressor (p=0.003) in multivariate logistic regression, ll. 186-188.*

-The abstract conclusions and main discussion section note that measures of AWC were significant predictors for severe COVID-19 outcomes. How were measures for statistical *significance* of ROC AUC performed? I may be missing it, but I see no mention of this in the methods.

*ROC analysis was omitted in the revised manuscript.*

-Please review the text carefully for typos and grammatical issues (e.g. line 53 “December 31rd”, line 64 “cardial mortality”, etc).

*The text was carefully reviewed for typos and grammatical issues.*

Comments of Reviewer #2:

-First of all, the authors don’t show the sufficient data and analyses to reach their conclusion. And the authors give too great an emphasis to the result. They should tone the conclusion down because there are many limitations. If authors want to mention the association between some parameters and outcomes, they should adjust confounding factors by controlling inclusion or multivariable analyses.

*Statistical methods were revised and a larger patient population was included. Within multivariate logistic regression AWC was the only significant regressor (p=0.003) after including the control variables patient age and sex, see ll.186-188. Limitations are added, see ll. 252-258.*

-And, basically, in retrospective study, it is not proper to use the word “predictor”. Regarding writing, the English language and sentence structures/styles in this manuscript seem to be improper as the manuscript submitted to academic journals. In several parts, the context between sentences are logically strange and not connected. English is not my native language and it is the reason why I always ask professionals in drafting to correct my manuscripts. I don’t think every non-native English speaker should do the same. However, journal submitters should take it into consideration according to an individual level of English and writing skill.

*The manuscript was revised for typos and grammatical issues. Additionally, large parts of the manuscript were omitted, rearranged and rewritten to clarify the logical train of thoughts. Concerning the term “predictor”: our study remains retrospective. However, multivariate logistic regression identified AWC as a highly significant regressor which is intended to serve as a predictor of severe COVID-19. In this context the term “predict” is commonly employed in scientific literature.*

-Introduction is too long. The authors should focus on the topic relevant to the main purpose of the study with brief summary of the general information about the target disease. Many of sentences seem to be unnecessary to discuss the authors’ topic. Because of too long introduction, it is not clear why the authors think AWC and Agatston score may be useful for detect high risk patients with Covid-19. The authors should logically guide readers on the way to their hypothesis and the purpose of their study in the introduction.

*Introduction was largely rearranged, shortened and rewritten.*

-Substantial selection bias is thought to exist. How many patients were originally screened for SARS-CoV-2? What was the indication for LDCT in the authors’ institutions.

*Indication for LDCT at our institutions was added in methods section, see ll. 106-107. The authors are aware that the portion of patients receiving LDCT doesn’t represent the general population of patients with suspected COVID-19. Indication for imaging is defined by careful consideration of cost against benefit of LDCT. In most patients with COVID-19 imaging is not indicated. However, for clinically moderate, severe and worsening disease LDCT is recommended. Hence the patient population of our study should comprise all moderate and severe COVID-19 cases which allows for our main goal: discrimination of severe against moderate cases.*

-It is not proper way for the author to justify the observation period by other publications. And the authors misunderstood. When the value is described as medial 18.5 days (15.0 – 22.0), the numbers in parenthesis show inter-quartile range in general, not minimum and maximum.

*The respective passage was adjusted, see inclusion criteria, ll. 108-113.*

-I am not a specialist in statistics. But, I found statistical analysis seems to be wrong in some parts. For example, Wilcox rank analysis is basically used for non-parametric variables, and which values should be described as median and inter-quartile range.

*Statistical methods were carefully revised. AWC and patient age are compared by a two-sided T-test, patient sex by Chi-square test, see l. 174.*

*-*Basic characteristics don’t include sufficient information. The authors should include at least comorbidities.

*See limitations of our study, ll. 253-256: “Comorbidities of patients were not respected, since our aim was to model a prediction solely based on imaging and generic patient data. This recognizes that a comprehensive clinical examination and anamnesis might not be achievable for every individual during the pandemic.”*

-Some parts of the discussion don’t seem to be related to this study. The authors should focus on the main topic in this study. What is the superiority of this study to others? What is the clinical implication?

*Discussion was shortened and rearranged. The superiority of a short-term stable parameter like AWC against a rapidly evolving parameter such as “involvement of the lung” is deducted in ll. 228-235. Clinical impaction is underlined in the conclusion, see ll. 261-270.*

-Please add the limitations of this study.

*Limitations are added, see ll. 252-258.*

-Many contents in the conclusion should be included in the discussion. Conclusion should be more concise and focused on the most important message delivered from this study.

*Conclusion and discussion are rearranged and shortened.*

Minor comments:

Introduction

-“By the time of submission of this article in May 2020” is not the style for academic journals. Please consider to change.

*The introduction was largely rearranged and rewritten.*

Methods

-Please describe the name and location of involved institutions.

*See ll. 102-103.*

-Please change the description from “by an M.D.” to “single observer (initial)”.

*See l. 116.*

-When was LDCT performed? At the same day with test for SARS-CoV-2?

*RT-PCR test and LDCT were performed in the same context, mostly on admission to the hospital. However, arteriosclerotic burden doesn’t change in the short-term and thus short-term delay of imaging doesn’t influence the magnitude of AWC.*

-The authors used the vendor’s software of Phillips for quantification of calcium. Is this vendor independent software? Can this software analyze the images acquired by other vendor’s machines?

*Each of the established CT vendors provides a software for calcium quantification. Besides, non-commercial solutions are available. The HeartBeat-CS software by Philips Healthcare can process images acquired by other vendor’s scanners. In our study we imported DICOM files from the Siemens scanner in center 2 to the Philips software.*

-There doesn’t seem to be the description how to measure totals of AWC.

*See ll. 136-146 for a detailed description of the measurements.*

-Table 1 should include p value. Why didn’t the authors use T-test for comparison?

*Statistical methods were revised and two-sided T-test is employed for comparison.*

I hope this letter comprehensively answers the reviewers’ comments and you further consider our manuscript for publication.

Sincerely,

Philipp Fervers
